# A Randomized Controlled Study of Fuzheng Huayu Capsule for Prevention of Esophageal Variceal Bleeding in Patients with Liver Cirrhosis

**DOI:** 10.1155/2013/534960

**Published:** 2013-05-21

**Authors:** Jie Gu, Qin Zhang, Dongying Xue, Hong Cai, Lieming Xu

**Affiliations:** ^1^Shuguang Hospital and Institute of Liver Diseases, Shanghai University of Traditional Chinese Medicine, Zhang Heng Road, Shanghai 201203, China; ^2^Shanghai Public Health Center, Shui Dian Road, Shanghai 200083, China; ^3^Putuo Hospital Affiliated to Shanghai University of Traditional Chinese Medicine, Lan Xi Road, Shanghai 200062, China; ^4^Xiamen Hospital of Traditional Chinese Medicine, Xian Yue Road, Xiamen 361009, China; ^5^Key Laboratory of Liver and Kidney Diseases, Ministry of Education, Cai Lun Road, Shanghai 201203, China

## Abstract

To elucidate the role of Fuzheng Huayu Capsule, a herbal formula, in the prevention of esophageal variceal bleeding in cirrhotic patients, a multicenter randomized and placebo-controlled trial was carried out. One hundred forty-six cirrhotic patients with esophageal varices were enrolled to compare the probability of upper gastrointestinal bleeding and survival between Fuzheng Huayu Capsule group and controlled group for the duration of 2 years. The results demonstrated that the FZHYC could effectively reduce the risk of variceal bleeding and improve survival rates for cirrhotic patients with varices, especially the combination of the capsule and Propranolol, which presented a better effect; FZHYC could reduce the varices size in patients with small ones. Its effect may be related to the amelioration of hepatic fibrosis.

## 1. Background

Esophageal variceal bleeding is a common and severe complication of liver cirrhosis and is a major cause of death. Although there is a great advance in the therapy of internal medicine and surgery, the prognosis of portal hypertension has not been remarkably improved. The prevalence of variceal hemorrhage is approximately 5% to 15% yearly, and early variceal rebleeding has a rate of occurrence of 30% to 40% within the first 6 weeks. More than 50% of patients who survive from first bleeding episode will experience recurrent bleeding within 1 year [1]. Therefore, prevention of esophageal variceal bleeding and rebleeding is crucial to reduce the risk of bleeding, increasing survival rate, and improve prognosis. 

Currently, *β*-blocker Propranolol is the first choice for prevention of esophageal and gastric variceal bleeding by reducing portal pressure [2]. However, there is a debate whether *β*-blocker could be used in patients with small varices. In addition, some patients are intolerant to *β*-blocker due to its contraindication and side effects. The combination of *β*-blocker and other drugs have been explored and applied in clinical practice so as to improve efficacy and reduce side effects. Fuzheng Huayu Capsule (FZHYC), a traditional Chinese herbal formula, has demonstrated the effect of antifibrosis and lower portal pressure in patients with chronic hepatitis B [3]. Thus, we conducted a clinical study to evaluate the effect of the capsule on the incidence of esophageal variceal bleeding and survival rate in cirrhotic patients. 

## 2. Methods

The study was an investigator-initiated, randomized, double-blind, placebo-controlled, and multicentered clinical trial conducted at four sites. The protocol was approved by Shuguang Hospital Ethics Committee, and all patients were given written informed consent. 

### 2.1. Patients

Because of first exploration for FZHYC treatment on prevention of variceal bleeding, there were not any basic parameters provided as references. Thus we conducted the small clinical trial and 30 subjects are designed to be recruited in each group. We recruited the patients from eligible inpatients and outpatients. Patients were enrolled between March 2003 and March 2004 and were followed until June 2006 in Shuguang Hospital, Shanghai Public Health Center, Putuo Hospital and Xiamen Hospital. The diagnosis of cirrhosis was either by liver biopsy or clinical history. The age range was between 18 and 70 years old. Eligible patients had HBV-induced cirrhosis with portal hypertension and gastroesophageal varices, as defined by endoscopy. They did not have peptic ulcer or hemangioma and did not have the treatment of endoscopic variceal ligation, endoscopic sclerotherapy, TIPS, or any surgical procedure for portal hypertension. All patients were not receiving antiviral therapy because their hepatitis B viral replication was lower or they refused antiviral therapy for some reasons. The patients were screened for the presence of HCC prior to entry into the study by measurement of AFP and ultrasonographic screening.

Exclusion criteria included serious liver disease such as (1) Child-Pugh score > 12; (2) ALT > 10 × ULN; (3) with hepatorenal syndrome; (4) with splenic or portal vein thrombosis; (5) administration of any drug or procedure affecting splanchnic hemodynamics or portal pressure; (6) contraindications to *β*-blocker therapy such as second and third degree atrioventricular block, sinus bradycardia, systolic blood pressure < 85 mmHg, asthma, or heart failure. 

### 2.2. Medicine for Intervention

FZHYC was the herbal extraction composed of Dan Shen (Radix Salviae Miltiorrhizae), Chong Cao (*Paecilomyces hepiali* Chen & Dai), Tao Ren (Semen Persicae), Jiao Gu Lan (Gynostemma Pentaphyllum), Song Hua Feng (*Pinus armandii* Franch), and Wu Wei Zi (Fructus Schisandrae Chinensis). The study drug was manufactured by Shanghai Modern Pharmaceutical Company. The composition of 5 capsules (0.3 g per capsule) is the same as that of 24 g raw herbs. The dose was 15 capsules per day and orally administrated at 3 times. FZHYC is recommended to intake directly after a meal, for some patients experiencing discomfort in the abdominal area in fasting condition.

The placebo was composed of stir-fried wheat powder which was also manufactured by Shanghai Modern Pharmaceutical Company. The placebo had an identical appearance in the aspects of dosage, color of the contents, and packaging. 

The starting dose of Propranolol was 10 mg per day and was increased by 10 mg until one of the following occurred: the resting heart rate was reduced by 25% from the baseline value or to 55 beats per minute.

Patients in each group were treated based on treatment guideline, for vitamins, liver protection drugs, and diuretics were applied depending on the condition of the patients. During the intervention period, other drugs for anti-fibrosis or affecting portal pressure were not administrated. 

### 2.3. Randomization

The present study is randomized, double blind, controlled, and multi-centered. According to the degree of esophageal and gastric varices, patients were stratified as 2 levels: small level and moderate/severe level. The randomization code was generated by SAS software for each subject.

For patients with small varices, the trial was double blinded. The patients were randomly assigned to 2 groups: one received FZHYC while the other received placebo. For patients with moderate and severe varices, the trial was single blinded. The patients were divided into 3 groups: FZHYC group, Propranolol group, and Combination group in which patients received both drugs. It was designed that 30 subjects would be recruited in each group for a total of 150 subjects in the present study.

### 2.4. Followup

Patients were assessed clinically at baseline, one and three months after randomization and every three months thereafter. At each visit, the heart rate, pill count, and occurrence of adverse events were investigated and blood was obtained for hematological and biochemical measurements. All adverse events, regardless of their possible association with the disease or study treatment, were recorded. An adverse event was judged severe, if it was considered to endanger the health or safety of the patient.

### 2.5. End Points

The clinical intervention period lasts 2 years and the followup would stop once the end points occur. 

The primary end point was esophageal or gastric variceal bleeding. All patients were instructed to go to the hospital whenever they experienced melena or bleeding. Esophageal or gastric variceal bleeding was defined as any hematemesis or melena in a patient in whom endoscopy showed active bleeding from an esophageal or gastric varix, an esophageal or gastric varix with an adherent clot, or varices but no other source of bleeding. In addition, acute, clinically significant bleeding as a result of portal hypertensive gastropathy was considered a primary end point. 

Secondary end points were the development of primary hepatocellular carcinoma, liver transplantation, or death. 

### 2.6. Statistical Analysis

An intent-to-treat strategy was used in the analysis of the results. Quantitative variables were presented as means with SDs. A student's *t-*test was used to compare differences of two groups in a small level. ANOVA was used to compare differences among groups in moderate and severe levels. Categorical variables were compared using Fisher's exact test. The Kaplan-Meier method was used to calculate probabilities of survival and freedom from variceal bleeding and differences were compared by log rank test. 

Statistical significance was established at a *P* value less than 0.05. SPSS statistical software package was used for the analysis.

## 3. Results

### 3.1. Patients

**Figure 1:**
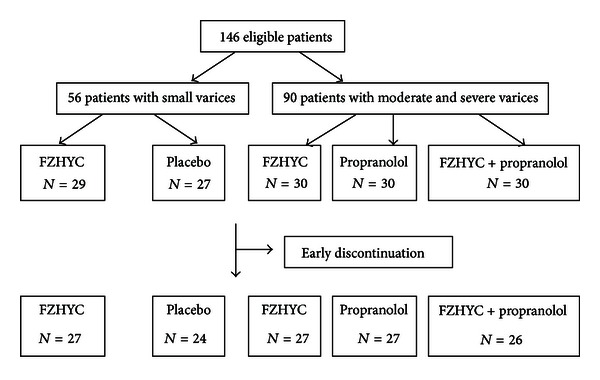
Enrollment of patients included in the study.

(1) Fifty-six patients met criteria for inclusion of small varices level (29 patients in FZHYC group and 27 patients in placebo group) in the follow-up study. The patients in 2 groups were similar in demographic characteristics of age, gender, Child-Pugh class, Child-Pugh score, Platelet count, Prothrombin time, albumin and total bilirubin concentrations, and inner diameter of portal vein. The clinical characteristics were no statistically different in baseline between 2 groups (*P* > 0.05). Three patients (1 patient in FZHYC group, 2 patients in placebo group) withdrew because they felt their condition had not improved; 2 patients (1 patient for each group) lost followup because their address had changed (Table 1 and Figure 1). 

(2) Ninety patients were recruited in the level of moderate and severe varices (30 patients for each group). There were statistically no differences of age, gender, Child-Pugh Class and score, and inner diameter of portal vein in baseline among 3 groups (*P* > 0.05). Only Prothrombin time in the FZHYC group (15.14 ± 1.47 s) was shorter than it was in the Propranolol group (16.79 ± 2.32 s, *P* < 0.05). The intervention drug was discontinued early in 10 patients (3 patients in FZHYC group, 3 patients in Propranolol group and 4 patients in the Combination group). Among these patients there were 5 patients unsatisfied with the efficacy, 1 patient emigrated abroad, one pursued a job outside Shanghai, one lost contact due to his relocation, and 2 patients could not tolerate the side effects by Propranolol (Table 2 and Figure 1). 

### 3.2. Primary End Point

#### 3.2.1. Patients with Small Varices Level

**Figure 2:**
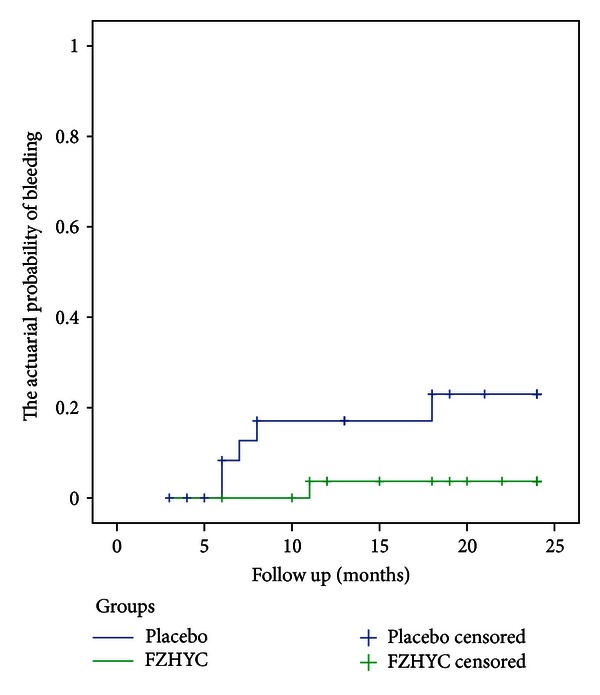
Actuarial probability of esophageal variceal bleeding in small varices level.

A total of 6 patients reached the primary end point of esophageal variceal bleeding: 1 of 29 patients in the FZHYC group and 5 of 27 patients in the placebo group. The actuarial probability of esophageal variceal bleeding was significantly different between 2 groups (3.7% versus 23.0%, *P* = 0.0422) (Figure 2). 

#### 3.2.2. Patients with Moderate and Severe Varices Level

**Figure 3:**
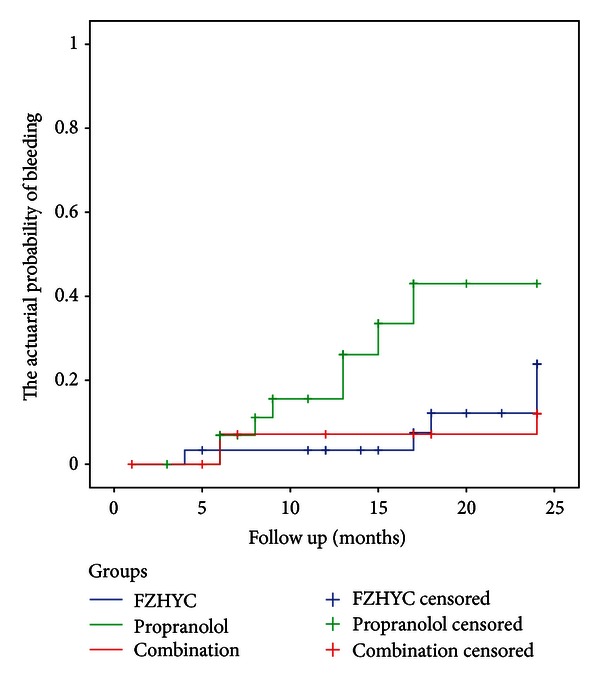
Actuarial probability of esophageal variceal bleeding in moderate and severe varices level.

Five patients in FZHYC group, 8 patients in Propranolol group, and 3 patients in the Combination group had esophageal variceal bleeding during their followup. Compared to the Propranolol group (43.0%), there were significant differences in the actuarial probability of esophageal variceal bleeding in FZHYC group (23.9%, *P* = 0.0131) and the Combination group (12.4%, *P* = 0.0086). There was a lower trend of rate of variceal bleeding in Combination group than in FZHYC group, but no significant difference (*P* = 0.3876) (Figure 3).

### 3.3. Secondary End Points

#### 3.3.1. Survival

For patients with small varices, there was 1 patient who died from liver failure in each group. There was no significant difference in the actuarial probability of survival (95.24% in FZHYC group, 95% in the placebo group, *P* = 0.7919), suggesting FZHYC has no effect on increasing the survival of the cirrhotic patients with small varices.

**Figure 4:**
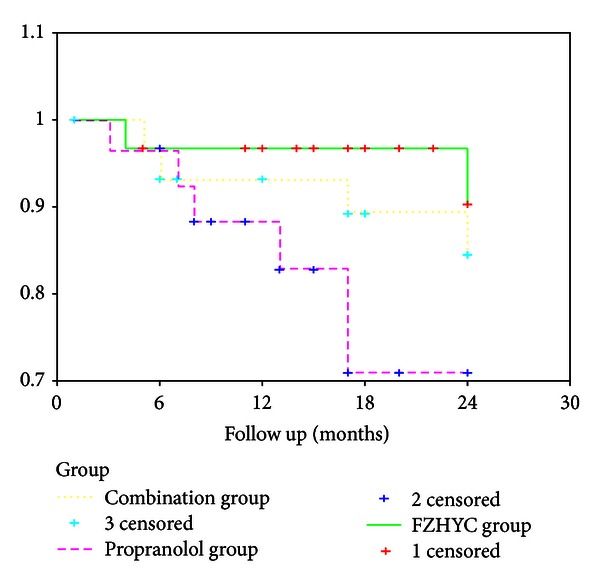
Actuarial probability of survival on patients with moderate and severe varices.

For cirrhotic patients with moderate and severe varices, 2 patients in FZHYC group, 5 patients in Propranolol group, and 4 patients in the Combination group died during the followup. The actuarial probability of survival in the FZHYC group was higher than that in the Propranolol group (90.22% versus 70.92%, *P* = 0.0449) demonstrating the herbal capsule may improve the survival of cirrhotic patients with moderate and severe varices. There were no significant differences in the actuarial probabilities of survival between the FZHYC group and the Combination group (90.22% versus 84.53%, *P* = 0.4691) and between the Propranolol group and the Combination group (70.92% versus 84.53%, *P* = 0.2298) (Table 3 and Figure 4).

### 3.4. Effect of FZHYC on Child-Pugh Score

At the baseline of the FZHYC group and the placebo group in the small varices level, there was no significant difference of Child-Pugh score. The score was decreased in the FZHYC group but increased in the placebo group at the time points of months 6, 12, 18, and 24 with the comparison of the baseline (Table 4). The difference, however, was not remarkable at each time point between 2 groups. It was indicated that the FZHYC only has a tendency to decrease Child-Pugh scores in cirrhotic patients with small esophageal varices. 

For patients with moderate and severe varices, there was no significant difference of Child-Pugh scores among the FZHYC group, the Propranolol group, and the Combination group at the baseline. By the comparison of the Child-Pugh score between the baseline and after treatment of 6th month or 12th month, it was shown that the FZHYC decreased the score. The difference of the scores was significant both in the FZHYC group or the Combination group to the Propranolol group after treatment (*P* < 0.05) (Table 5).

### 3.5. Effect of FZHYC on Development of Small Varices

Some patients with small varices were willing to be reexamined by endoscopy after 24 months of treatment. There was a significant difference of esophageal varices degree in the FZHYC group compared to the placebo group after 24 months of treatment (*P* = 0.014) (Table 6).

## 4. Discussion

It is reported that esophageal varices will not spontaneously disappear once they are formed [4, 5]. Although some experimental study has shown that early application of *β*-blocker can prevent the formation of collateral circulation in the state of portal hypertension [6], significantly lowering the risks in the variceal size from small to severe [7]. Currently, only for patients with small varices and red signs or advanced liver failure (Child-Pugh C), nonselective *β*-blockers are indicated for primary prophylaxis of bleeding [2]. 

Both nonselective-blockers and endoscopic therapy are effective in preventing first variceal bleeding for patients with cirrhosis and moderate/large varices that have not bled; therefore the decision should be based on patient characteristics and preferences, local resources, and expertise [2]. The ideal treatment would be one that is universally effective, safe, freely available, easy to administer, and inexpensive. Considering the factors of the efficacy, varices size, the patients' acceptance, and medical cost in China, pharmacological therapy is applied to prevent first variceal bleeding in this study.

*β*-blocker could reduce the risk of first bleeding by 40%~50% in patients with moderate and severe varices [8]. The present study showed that the actuarial probability of first variceal bleeding in patients with moderate and severe varices was 56.99%, in line with other relevant studies. 

FZHYC, a Chinese herbal formula, has been developed as a new intervention to treat liver fibrosis in the past 20 years. While the active components of FZHYC have not been fully clarified, a great number of the active components can be directly absorbed into the blood after oral administration of YZHYC in our previous studies: amygdalin from Semen Persicae; salvianolic acid B and tanshinol from Radix Salvia Miltiorrhizae; Gypenosides from Gynostemma Pentaphyllum; schisandrin, schizandrol B, schisantherin A, and schisandrin B from Schisandra Chinensis.

According to Practice guidelines for the diagnosis and treatment of gastroesophageal variceal hemorrhage endorsed by the American Association for the Study of Liver Diseases (AASLD, 2009), some clinical trials quote the incidence of first variceal hemorrhage of patients, who were with cirrhosis and small varices that have notbled, was 7% to 14% over 2 years or 37% at 3 years. However, our 2-year-long clinical study showed that esophageal variceal bleeding occurred in 5 of 27 patients with small varices in the placebo group. The risk of bleeding was 23% at 2 years. But the actuarial probability of variceal bleeding was only 3.7% in the Fuzheng Huayu group, which was significantly lower than the placebo group (*P* = 0.0422). However, there was no significant difference of survival rate between both groups. All cirrhotic patients recruited in the present study were induced by hepatitis B virus, thus their varices could not disappear spontaneously in theory. After 24 months of clinical observation, endoscopy was performed to patients with small varices. Eight patients did not present varices in the Fuzheng Huayu group, while only 1 patient in the placebo group showed such improvements, and there was significant difference between both groups. Because the placebo group also received regular treatment, the improvement might be the result of regular treatment. By comparison, the improvement in the Fuzheng Huayu group was more significant. FZHYC could decrease the varices size and lowered the risk of bleeding.

FZHYC also prevented variceal bleeding in patients with moderate and severe varices. The actuarial probability of variceal bleeding was significantly lower and the actuarial probability of survival was significantly higher in the Fuzheng Huayu group than that in the Propranolol group. The Combination group presented a better effect than the other 2 groups. The present study showed that patients with the administration of the FZHYC had a better efficacy than the Propranolol group.

The mechanism of the FZHYC for preventing bleeding is different from Propranolol. Nonselective *β*-blocker reduces portal pressure through a reduction in portal venous inflow as a result of a decrease in cardiac output (*β*1-adrenergic blockade) and splanchnic blood flow (*β*2-adrenergic blockade) [9, 10]. Through the actions of anti-fibrosis [3, 11] and the nondecrease of cardiac output (the data not show), FZHYC could enhance hepatic blood circulation by increasing the blood flow volume and speed in the liver (the data not shown), thus lowering the risk of bleeding. 

FZHYC was originally designed to treat hepatic fibrosis. The pathological results from three previous clinical trials demonstrated that FZHYC markedly lessened inflammation in the liver, prevented the process of hepatic fibrosis induced by hepatitis B virus, and enhanced the degradation of hepatic fibrosis in patients with hepatic fibrosis [12–14]. 

*In vivo* and *in vitro* studies have revealed the mechanisms of FZHYC to treat hepatic fibrosis. That were as follows. (1) Protect hepatocytes. It could improve the liver function, decrease hepatic oxidative stress, and inhibit apoptosis of hepatocytes both in the clinical study and experimental study [15–17]. (2) Inhibit hepatic stellate cells (HSC) activation. It could inhibit PDGF-BB-stimulated proliferation of HSC, inhibit collagen secretion in a dose dependent manner, in particular collagen type I secretion and gene expression, and decrease TGF-*β*1 expression in activated HSCs [18, 19]. (3) Decrease the concentration of endothelin-1 (ET-1). 

No observable adverse effect on FZHYC was reported and there was no significant difference in blood and urine routines and ECG before and after treatment in a multicenter, randomized, double-blinded clinical study including 216 patients with hepatic fibrosis caused by chronic hepatitis B [11]. 

## 5. Conclusions

The FZHYC could effectively reduce the risk of variceal bleeding and improve survival rates for cirrhotic patients with varices, especially the combination of the capsule and Propranolol, which presented a better effect; the capsule could reduce the varices size in patients with small ones. Its effect may be related to the prevention of hepatic fibrosis, amelioration of liver function, and the decrease of ET concentration in the blood plasma. 

## Figures and Tables

**Table 1:** Baseline characteristics of the patients in small varices level.

Characteristic	FZHYC group (*n* = 29)	Placebo group (*n* = 27)
Age (yr)	49 ± 8	50 ± 8
Gender: M/F (*n*)	26/3	21/6
Child-pugh class (*n*)		
A	22	19
B	6	7
C	1	1
Child-pugh score	5.72 ± 1.22	6.18 ± 1.14
Platelet count (10^9^/L)	89.14 ± 47.62	76.67 ± 40.07
Prothrombin time (s)	15.15 ± 3.12	15.43 ± 3.02
Total bilirubin (mmol/L)	24.60 ± 8.91	27.07 ± 10.04
Albumin (g/L)	39.65 ± 6.07	36.58 ± 7.55
Inner diameter of portal vein (mm)	12.54 ± 0.92	12.65 ± 1.33

**Table 2:** Baseline characteristics of the patients in moderate and severe varices level.

Characteristic	FZHYC group (*n* = 30)	Propranolol group (*n* = 30)	Combination group (*n* = 30)
Age (y)	51 ± 9	50 ± 8	49 ± 8
Sex: M/F (*n*)	21/9	21/9	24/6
Child-Pugh class (*n*)			
A	19	16	17
B	10	12	10
C	1	2	3
Child-Pugh score	6.33 ± 1.26	6.71 ± 1.53	6.89 ± 1.95
Platelet count (×10^9^/L)	58.73 ± 28.17	55.79 ± 24.92	61.32 ± 19.92
Prothrombin time (s)	15.14 ± 1.47*	16.79 ± 2.32	15.99 ± 2.52
Total bilirubin (mmol/L)	29.12 ± 13.83	36.21 ± 23.15	34.77 ± 21.89
Albumin (g/L)	37.13 ± 5.53	35.71 ± 5.43	35.31 ± 6.89
Inner diameter of portal vein (mm)	13.41 ± 1.81	13.41 ± 1.47	13.09 ± 1.77
EV degree (*n*)			
II grade	22	20	21
III grade	8	10	9
Red sign	8	10	9
Gastric varices (*n*)	4	6	5
Portal hypertensive gastropathy (*n*)	13	8	12

*Compared to Propranolol group, it did not differ significantly (*P* < 0.05).

**Table 3:** Causes of death in patients with moderate and severe varices (*n*).

Groups (*n*)	Variceal bleeding (*n*)	Liver failure (*n*)	Hepatocellular carcinoma (*n*)
FZHYC group (30)	1	1	0
Propranolol group (30)	0	3	2
Combination group (30)	2	2	0

**Table 4:** Effect of FZHYC on Child-Pugh score in patients with small esophageal varices.

Groups (*n*)	0 m	6th m	12th m	18th m	24th m
FZHYC group (27)	5.72 ± 1.22	5.33 ± 1.00	5.27 ± 0.88	5.15 ± 0.49	5.11 ± 0.49
Difference		−0.37 ± 0.68	−0.27 ± 0.55	−0.20 ± 0.61	−0.23 ± 0.56
Placebo group (24)	6.18 ± 1.14	6.33 ± 1.65	6.17 ± 1.55	6.14 ± 1.23	6.11 ± 1.53
Difference		0.14 ± 1.16	0.18 ± 1.79	0.15 ± 1.34	0.62 ± 1.50

**Table 5:** Changes of Child-Pugh score in patients with moderate and severe esophageal varices.

Groups (*n*)	0 m	6th m	12th m
FZHYC group (30)	6.33 ± 1.26	5.71 ± 0.89	5.88 ± 1.09
Difference		−0.5 ± 1.10*	−0.44 ± 1.29*
Propranolol group (30)	6.71 ± 1.53	6.52 ± 1.28	6.92 ± 1.65
Difference		−0.18 ± 0.91	0.50 ± 1.61
Combination group (30)	6.89 ± 1.95	6.12 ± 1.36	5.81 ± 1.28
Difference		−0.58 ± 1.24*	−0.83 ± 1.52*

*Compared to Propranolol group, the difference of Child-Pugh scores before and after the treatment differed significantly (*P* < 0.05).

**Table 6:** Difference of developing small varices after 24 months of treatment.

Groups	*n*	Number of varices (*n*)	Number of changes (*n*)	Enlarged varices (*n*)
FZHYC group	15	8	5	2
Placebo group	9	1	3	5
